# V-ATPase: a master effector of E2F1-mediated lysosomal trafficking, mTORC1 activation and autophagy

**DOI:** 10.18632/oncotarget.4812

**Published:** 2015-08-11

**Authors:** Nathalie Meo-Evoli, Eugènia Almacellas, Francesco Alessandro Massucci, Antonio Gentilella, Santiago Ambrosio, Sara C. Kozma, George Thomas, Albert Tauler

**Affiliations:** ^1^ Departament de Bioquímica i Biologia Molecular, Facultat de Farmàcia, Universitat de Barcelona, 08028 Barcelona, Catalunya, Spain; ^2^ Laboratory of Cancer Metabolism, IDIBELL, Hospital Duran i Reynals, 08908 L'Hospitalet de Llobregat, Barcelona, Catalunya, Spain; ^3^ Departament d'Enginyeria Química, Universitat Rovira i Virgili, 43007 Tarragona, Catalunya, Spain; ^4^ Unitat de Bioquímica, Dep. Ciències Fisiològiques II, Facultat de Medicina, Campus Universitari de Bellvitge - IDIBELL, Universitat de Barcelona, 08908 L'Hospitalet de Llobregat, Barcelona, Catalunya, Spain; ^5^ Laboratory of Cancer Metabolism, Institut Català d'Oncologia, Hospital Duran i Reynals, 08908 L'Hospitalet de Llobregat, Barcelona, Catalunya, Spain; ^6^ Division of Hematology and Oncology, Department of Internal Medicine, College of Medicine, University of Cincinnati, Cincinnati, Ohio 45267, USA

**Keywords:** E2F1, v-ATPase, mTORC1, autophagy, lysosomes

## Abstract

In addition to being a master regulator of cell cycle progression, E2F1 regulates other associated biological processes, including growth and malignancy. Here, we uncover a regulatory network linking E2F1 to lysosomal trafficking and mTORC1 signaling that involves v-ATPase regulation. By immunofluorescence and time-lapse microscopy we found that E2F1 induces the movement of lysosomes to the cell periphery, and that this process is essential for E2F1-induced mTORC1 activation and repression of autophagy. Gain- and loss-of-function experiments reveal that E2F1 regulates v-ATPase activity and inhibition of v-ATPase activity repressed E2F1-induced lysosomal trafficking and mTORC1 activation. Immunoprecipitation experiments demonstrate that E2F1 induces the recruitment of v-ATPase to lysosomal RagB GTPase, suggesting that E2F1 regulates v-ATPase activity by enhancing the association of V_0_ and V_1_ v-ATPase complex. Analysis of v-ATPase subunit expression identified B subunit of V_0_ complex, ATP6V0B, as a transcriptional target of E2F1. Importantly, ATP6V0B ectopic-expression increased v-ATPase and mTORC1 activity, consistent with ATP6V0B being responsible for mediating the effects of E2F1 on both responses. Our findings on lysosomal trafficking, mTORC1 activation and autophagy suppression suggest that pharmacological intervention at the level of v-ATPase may be an efficacious avenue for the treatment of metastatic processes in tumors overexpressing E2F1.

## INTRODUCTION

The E2F1 transcription factor is over-expressed in numerous human cancers, including lung, breast and hepatocellular carcinomas, as well as Sporadic Burkitt's Lymphomas [1–4]. The E2F1 signature is also strongly associated with invasive tumor progression of breast and bladder tumors [2, 5]. In the past, the major role reported for E2F1 in cancer progression was its activation of cell cycle progression. Activation of E2F1 is sufficient to irreversibly commit cells to undergo DNA replication by transcriptional activation of a number of genes required for the G1/S transition and the coordination of mitosis [6, 7]. However, it is now evident that other biological processes associated with proliferation and malignant transformation are also regulated by E2F1, including cell growth, autophagy, invasiveness and metastasis [2, 8–11]. Despite the impact of these processes on cancer progression, the molecular mechanisms by which E2F1 regulates these responses are still unknown.

Previously, we reported that E2F1 regulates cell growth, increase in cell size, through activation of mTORC1, a major regulator of protein synthesis and autophagy [8]. Consistent with this observation, tumors from transgenic mice in which E2F1 is overexpressed possess high mTORC1 activity, suggesting that the effects of E2F1 on tumorigenesis may be in part mediated through mTORC1 [4]. The mechanism by which E2F1 regulates mTORC1 remains unknown. Earlier we showed that E2F1 drives mTORC1 activation independently of the canonical PI3K/Akt/TSC1-TSC2 pathway and demonstrated that, after E2F1 activation, mTORC1 co-localizes with the lysosomes [8]. Translocation of the mTORC1 complex to the lysosomes has been described as the mechanism by which mTORC1 interacts with and is activated by the small GTPase Rheb [12]. In parallel, amino acids activate a second family of GTPases, the Rags, which promote the translocation of mTORC1 to the lysosomal surface, a process dependent on the vacuolar H^+^ adenosine triphosphatase ATPase (v-ATPase) [13].

Lysosomes are organelles that function as the main catabolic compartments of eukaryotic cells and play a key role in the degradation of the extracellular matrix (ECM) [14]. Several proteases that contribute to ECM degradation, such as cathepsin L, B and D, are directly or indirectly associated with lysosomes [15]. Tumors and invasive cancer cells, which are dependent on effective lysosomal function, often show alterations in lysosomal volume, composition and cellular distribution [16]. In turn, the functionality of lysosomes is dependent on v-ATPase, the proton pump responsible for lysosomes' intravesicular acidification [14]. V-ATPase is also essential for lysosomal trafficking and its inhibition blocks lysosomal maturation at an early endosomal stage [17, 18]. Intracellular lysosomal movement is achieved by the cooperation of microtubules, actin, and myosin filaments and requires the coordination of multiple signaling events including mobilization of Ca^2+^, phosphoinositides, small GTPases and kinesins [19, 20]. Interestingly, activation of mTORC1 by nutrients has been correlated with the presence of lysosomes at the cell periphery [21].

In this study, we set out to elucidate the underlying molecular mechanisms by which E2F1 mediates mTORC1 activation and autophagy repression in U2OS osteosarcoma cells. This led us to novel findings concerning the ability of E2F1 in regulating v-ATPase activity. By modulating v-ATPase activity, E2F1 re-localizes lysosomes to the cell periphery and provides a potential mechanism by which E2F1 drives invasiveness and metastasis.

## RESULTS

### E2F1 regulates lysosomal localization

**Figure 1:**
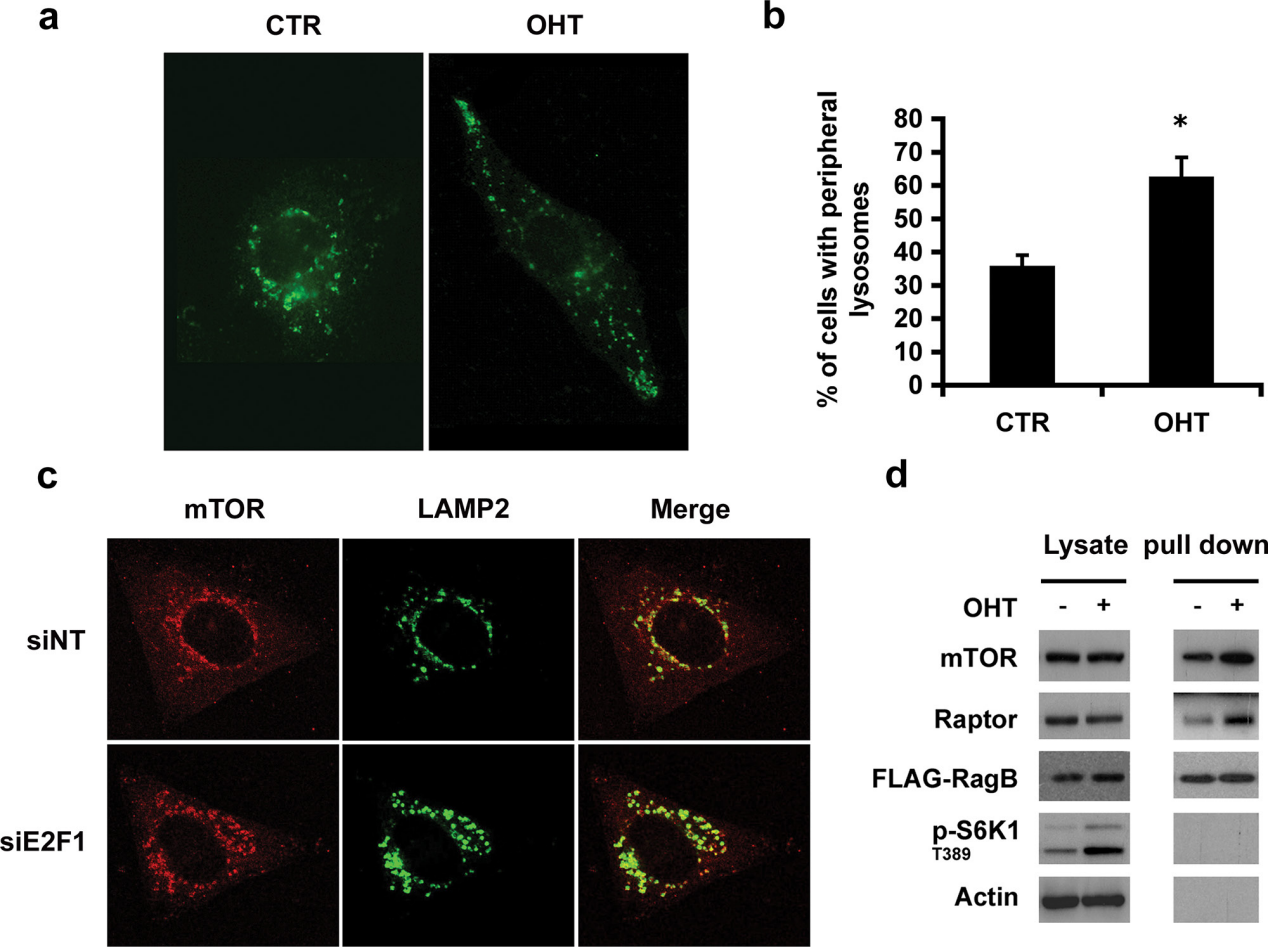
E2F1 induces lysosomal trafficking **a, b.** Serum-deprived ER-E2F1 U2OS cells were cultured in the absence (CTR) or in the presence of 4-hydroxitamoxifen (OHT) for 6 h. Immunofluorescence assay was performed as described in Materials and Methods using primary antibody against LAMP2. (b) Quantification of peripheral lysosomes localization is shown. Shown is mean ± S.E.M of 3 independent experiments. **c.** U2OS cells were transfected with non target siRNA (siNT) or E2F1 siRNA. Cells were serum-deprived and immunofluorescence was performed as described in Materials and Methods using primary antibodies against specified proteins at 48 h after transfection **d.** Stable transfected ER-E2F1/ FLAG-RagB U2OS cells were serum deprived and cultured in the absence (−) or in the presence of 4-hydroxitamoxifen (+) for 6 h. Proteins were crosslinked as described in Materials and Methods and immunoprecipitated using FLAG antibody. Expressions of the indicated proteins were determined by Western Blot analysis.

As mTOR co-localizes with the lysosome after E2F1 activation, we investigated whether E2F1 activity affected the distribution and trafficking of lysosomes [8]. For this study, we used the previously reported U2OS ER-E2F1 stable cell line [8]. The ER-E2F1 fusion protein is expressed in the cytosol and the addition of 4-hydroxitamoxifen (OHT) induces its translocation to the nucleus, where E2F1 regulates gene transcription. Analysis by immunofluorescence microscopy of endogenous LAMP2 protein showed that activation of E2F1 produced a striking change in intracellular distribution of LAMP2-positive vesicles. Whereas serum-deprived cells showed a predominantly perinuclear distribution of LAMP2-positive particles, activation of E2F1 induced the relocalization of LAMP2-positive particles to the cell periphery (Figures 1a and 1b). We also followed lysosomal mobility by live imaging employing time-lapse microscopy in cells expressing LAMP1-GFP, confirming the time-dependent redistribution of lysosomes to the cell periphery (Supplementary movie S1). Consistent with E2F1 regulating this response, siRNA depletion of endogenous E2F1 led to the accumulation of enlarged LAMP2 particles, which localized in the perinuclear region (Figure 1c). Interestingly, inhibition of lysosomal movement to cell periphery by knocking down E2F1 also produced a strong effect on the inhibition of cell migration (Supplementary Figure S1). The formation of the enlarged LAMP2 particles due to E2F1 depletion was also found in human lung adenocarcinoma cell line A549, suggesting that such phenomenon is not cell type-specific (Supplementary Figure S2a–S2c). Overall, the results show that E2F1 activation plays a role in redirecting lysosomes to the cell periphery.

In parallel, mTOR immunofluorescence largely overlapped with LAMP2 positive marker in E2F1-induced cells, most likely due to a direct interaction between mTORC1 and lysosomes (Supplementary Figures S3a and S3b). As the Rag GTPase family of proteins mediates the interaction of mTORC1 with lysosomes in the presence of amino acids, we hypothesized that E2F1 could induce the translocation of mTORC1 to lysosomes by a similar mechanism. To test this model, U2OS ER-E2F1 cells were stably transfected with FLAG-tagged RagB and the extent of RagB interaction with mTORC1 was measured in cells treated with the crosslinking reagent dithiobis (succinimidylpropionate) prior to lysis. The results from FLAG immunoprecipitation experiments demonstrated that the binding of mTOR and Raptor to RagB was increased after E2F1 activation, demonstrating the translocation of both proteins to the lysosomal compartment (Figure 1d). These results show that E2F1 promotes the translocation of mTORC1 into the lysosomes and increases the physical interaction of mTORC1 with the lysosomal protein RagB.

### Raptor, but not mTORC1 activity, is required for the E2F1-regulated lysosomal movement

**Figure 2:**
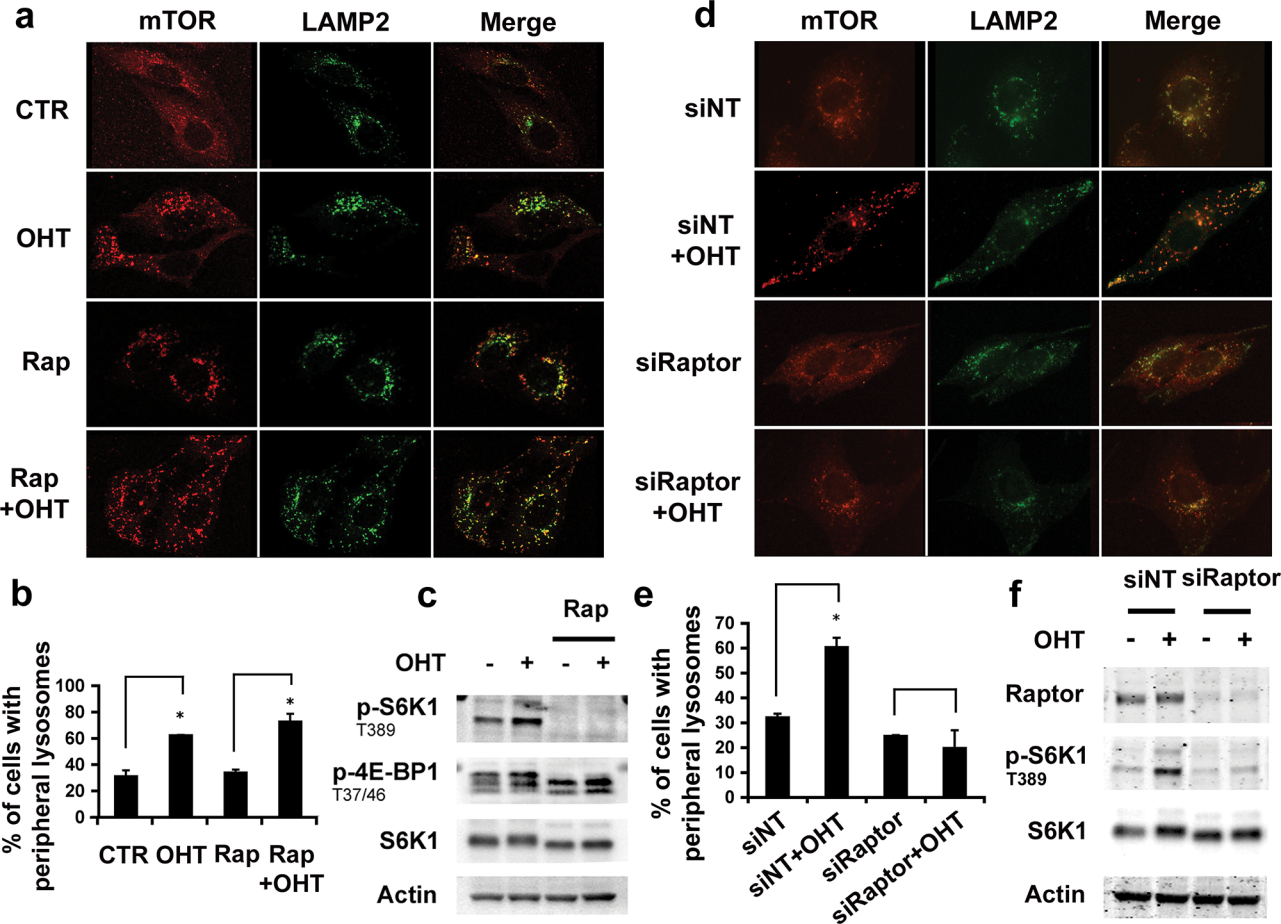
Effect of mTORC1 activity in lysosomal trafficking **a–c.** Serum-deprived ER-E2F1 U2OS cells were cultured in the absence (CTR) or in the presence of 4-hydroxitamoxifen (OHT) and treated or not with rapamycin (Rap) for 6 h. **d–f.** ER-E2F1 U2OS cells were transfected with non target siRNA (siNT) or Raptor siRNA (siRaptor) for 48 h. Cells were serum-deprived and treated (OHT) or not with 4-hydroxitamoxifen. (a, d) Immunofluorescence assay was performed as described in Materials and Methods using primary antibodies against showed proteins. Merge panels indicate the co-localization of antibodies signals. (b, e) Quantification of peripheral lysosomes localization is shown. Shown is mean ± S.E.M of 3 independent experiments. (c, f) Expression of the indicated proteins was determined by Western Blot analysis at 6 h after treatment.

As E2F1 promotes lysosomal trafficking, we asked whether E2F1-induced mTORC1 activation was implicated in this process. Analysis by immunofluorescence microscopy of LAMP2 and mTOR proteins showed that treatment with rapamycin, an mTORC1 allosteric inhibitor, did not alter E2F1-induced endogenous LAMP2 or mTORC1 relocalization to the cell periphery, despite abrogating mTORC1 basal activity, as measured by S6K1 T389 and 4E-BP1 T37/T46 phosphorylation (Figures 2a–2c). These data were also consistent with live-cell imaging of ectopically expressed LAMP1-GFP (Supplementary movie S2). To confirm that lysosomal localization was an mTORC1-independent process, we investigated the effect of E2F1 on lysosomal trafficking, in cells depleted of Raptor, an essential component of mTORC1. Depletion of Raptor impaired both mTORC1 activation, as measured by S6K1 T389 phosphorylation, and the movement of LAMP2 containing lysosomes to the cell periphery (Figures 2d–2f). Taken together, the results suggest that mTORC1 activity is not necessary for lysosomal trafficking induced by E2F1, but that the presence of Raptor is required for this response.

### E2F1 represses autophagy

**Figure 3:**
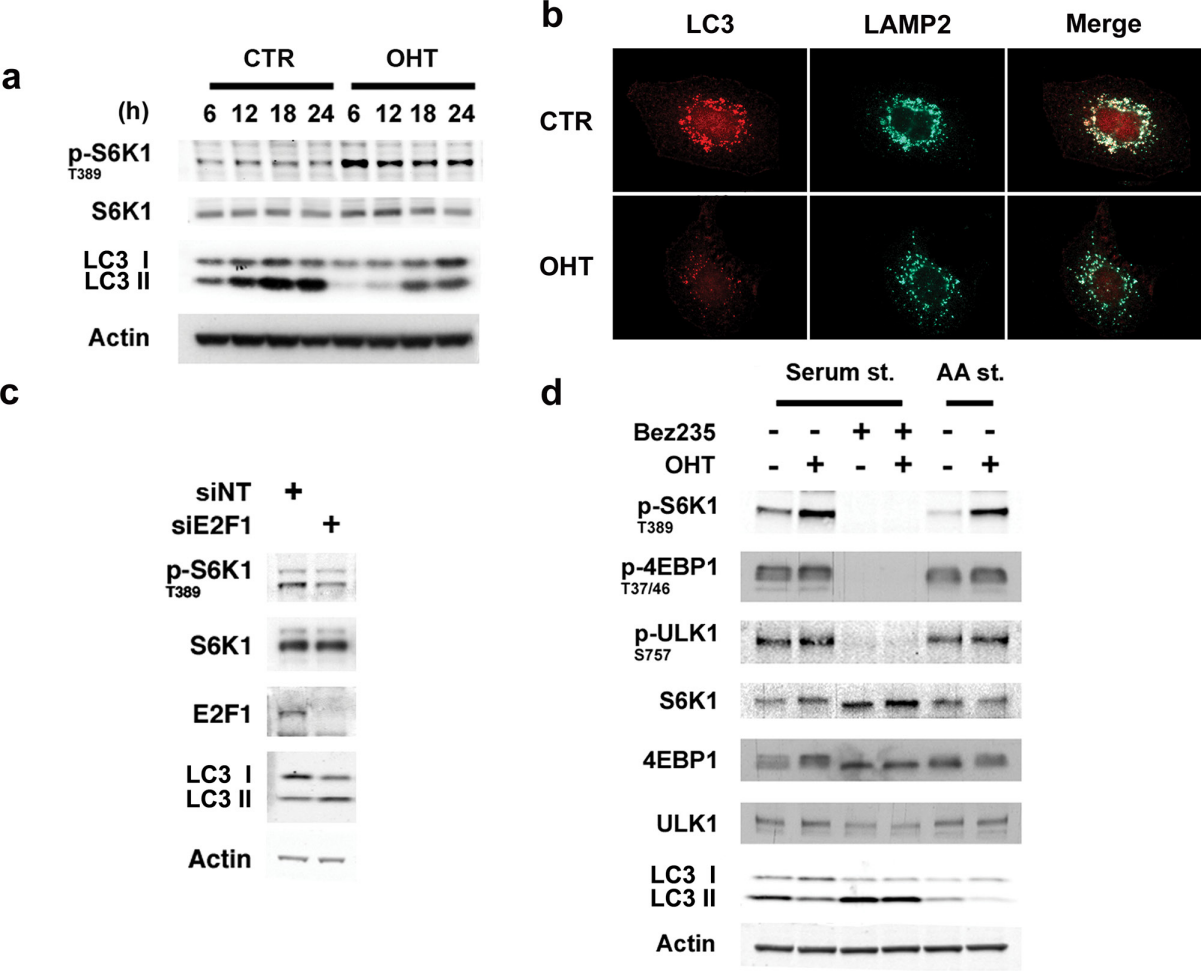
E2F1 represses autophagy **a, b.** Serum-deprived ER-E2F1 U2OS cells were cultured in the absence (CTR) or in the presence of 4-hydroxitamoxifen (OHT). (a) At showed times, expression of the indicated proteins was determined by Western Blot analysis. (b) Immunofluorescence assay was performed as described in Materials and Methods using primary antibodies against showed proteins at 6 h after treatment. **c.** U2OS cells were transfected with non-target siRNA (siNT) or E2F1 siRNA. for 48 h and serum-depleted for 15 h. Expression of the indicated proteins was determined by Western Blot analysis. **d.** Serum-deprived or amino acids/serum deprived ER-E2F1 U2OS cells were treated (+) or not treated (−) with 4-hydroxitamoxifen (OHT) in the presence (+) or in the absence (−) of Bez235. Expression of the indicated proteins was determined by Western Blot analysis at 6 h after treatment.

As E2F1 regulates lysosomal trafficking and mTORC1 activation, we reasoned it could play a role in controlling autophagy. To investigate the function of E2F1 on autophagy, cells were deprived of serum for 24 hours and the effect of E2F1 on the conversion of LC3-I to LC3- II was monitored [22]. In serum-deprived cells, we observed a time-dependent accumulation of LC3-II, a response that was markedly suppressed by activation of E2F1 (Figure 3a). Inhibition of LC3-II flux by the addition of the protease inhibitor leupeptin confirmed E2F1′s role as a repressor of autophagy (Supplementary Figure S4a). Consistent with these findings, upon serum starvation, LC3 and LAMP2 formed prominent punctae and localized throughout the perinuclear region, while in E2F1-induced cells, LC3 staining was more diffuse, less abundant, and LAMP2 vesicles were localized at the cell periphery (Figure 3b). Furthermore, in agreement with the autophagy repressor role of E2F1, E2F1 depletion by siRNA was associated with the accumulation of LC3-II (Figure 3c). The disseminated localization of LC3 found in E2F1-activated conditions could indicate that autophagosome formation was inhibited by E2F1, potentially through the activation of mTORC1 [23]. Consistent with this possibility, incubation with BEZ235, a dual mTOR/PI3K inhibitor, totally abrogated the negative effect of E2F1 on autophagy, as measured by the increase in LC3-II levels (Figure 3d). As expected, BEZ235 inhibited the phosphorylation of the mTORC1 targets S6K1, ULK1 and 4EBP1 (Figure 3d). Similar results were obtained in the presence of rapamycin (Supplementary Figure S4b). Thus, these findings argue that E2F1 is a negative effector of autophagy and suggest that the migration of lysosomes to the cell periphery as well as activation of mTORC1 could both contribute to this response.

### E2F1 enhances v-ATPase activity, which modulates lysosomal movements and mTORC1 activity

**Figure 4:**
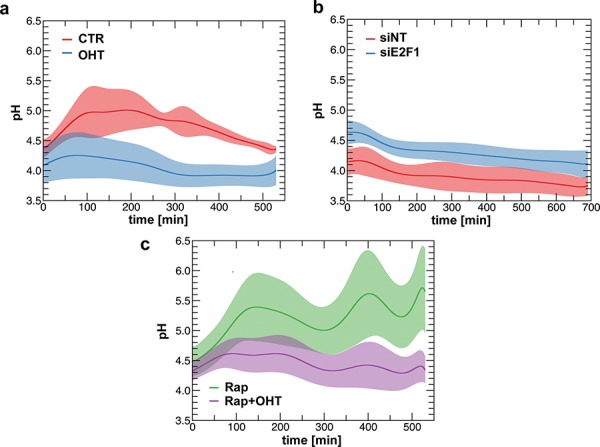
E2F1 regulates intra-lysosomal pH **a.** ER-E2F1 U2OS cells were loaded with dextran labeled with FITC and with Rhodamine B for 24 h. After 15 h of serum starvation, cells were treated (OHT) or not (CTR) with 4-hydroxitamoxifen and intra-lysosomal pH was measured as described in Materials and Methods. **b.** U2OS cells were transfected either with non target siRNA (siNT) or E2F1 siRNA and then loaded with dextran labeled with FITC and with Rhodamine B for 24 h. Intra-lysosomal pH was measured at 48 h after transfection as described in Materials and Methods. **c.** ER-E2F1 U2OS cells were loaded with dextran labeled with FITC and with Rhodamine B for 24 h. After 15 h of serum starvation, cells were treated (OHT) or not with 4-hydroxitamoxifen in the presence (Rap) of rapamycin, and intra-lysosomal pH was measured as described in Materials and Methods.

As v-ATPase has been functionally implicated in vesicular trafficking and mTORC1 signaling, we investigated whether E2F1 regulated v-ATPase activity [12, 24]. V-ATPase activity was measured by monitoring the pH of individual lysosomes after E2F1-induction. Lysosomes were loaded with dextran that was coupled to both fluorescein isothiocyanate (FITC) and to rhodamine, and then the changes in fluorescence intensity were followed by live-cell imaging system. FITC fluorescence decreases with acid pH, while rhodamine signal acts as a pH-independent control [25]. Using this approach, we found that the mean pH of the lysosomes decreases from 4.3–4.8, in serum deprived cells, to 3.8–4.2 in E2F1-induced cells, suggesting that v-ATPase activity is enhanced by the activation of E2F1 (Figure 4a). Consistent with this observation, activation of v-ATPase correlated with an increase in intra-cellular pH following E2F1 induction from ~6.94 to 7.07, as measured by using the pH-sensitive fluorescence dye 5-(and 6)-Carboxy SNARF-1 Acetoxymethyl Ester (Supplementary Figure S5). The potential role of E2F1 in the regulation of v-ATPase was further strengthened by siRNA-depletion of endogenous E2F1, which led to an increase in the lysosomal pH compared to the control (Figure 4b). The rise of intra-lysosomal pH could imply a decrease in lysosomal protease activity, in agreement with the finding above that depletion of E2F1 is associated with the accumulation of LC3-II (Figure 3c). To rule out the possibility that the change in v-ATPase activity could be mediated by mTORC1, we monitored the effect of rapamycin treatment on the intra-lysosomal pH. Even in the presence of rapamycin, intra-lysosomal pH values observed in E2F1-induced cells were lower than in non-induced cells, corroborating that mTORC1 activity was not required for the increased proton flux into lysosomes (Figure 4c).

**Figure 5:**
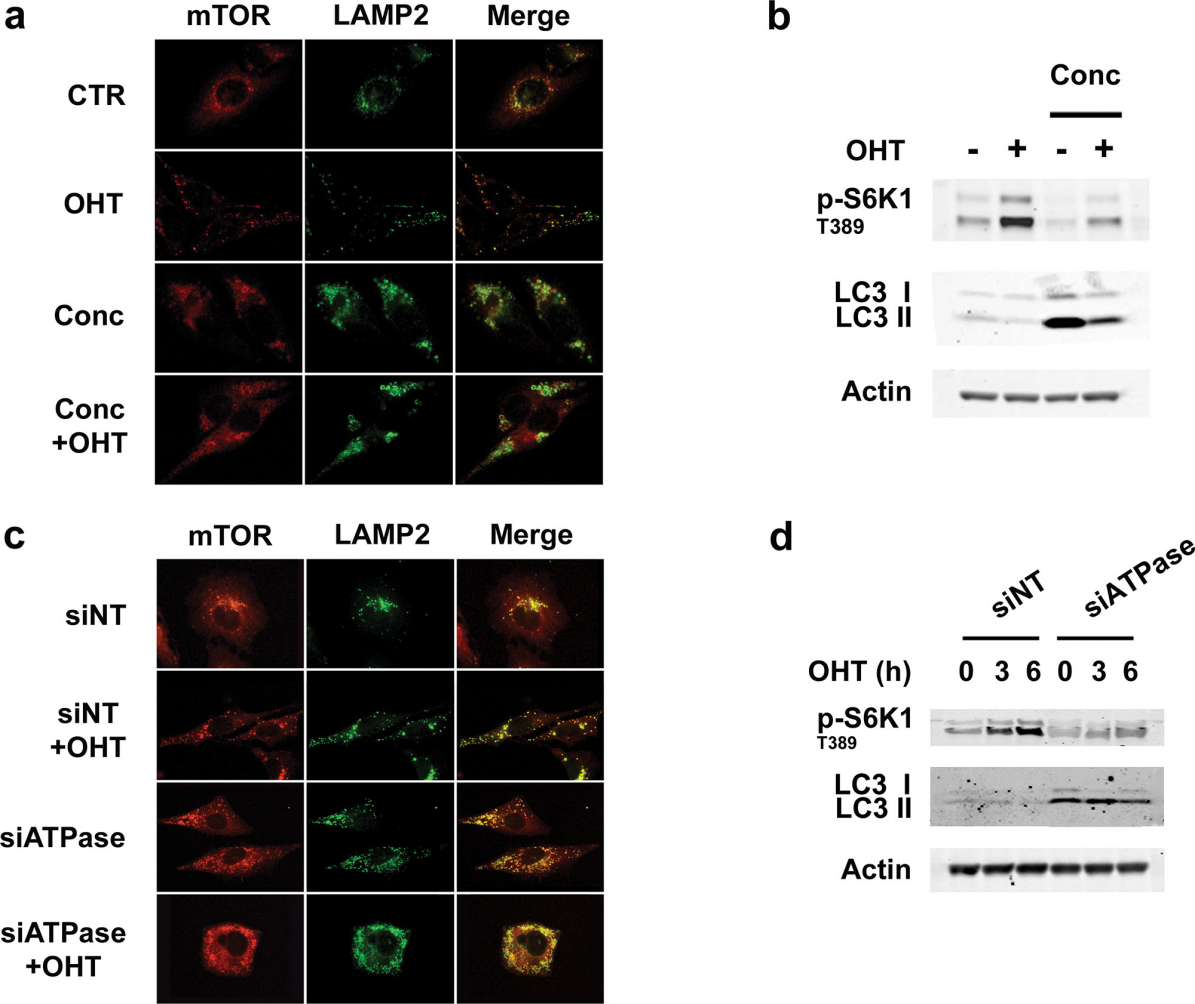
Inhibition of v-ATPase activity represses E2F1-induced mTORC1 activation and lysosomal trafficking **a, b.** Serum-deprived ER-E2F1 U2OS cells were cultured in the absence (CTR) or in the presence of 4-hydroxitamoxifen (OHT) with or without concanamycin (Conc). (a) At 6 h after treatment, immunofluorescence assay was performed as described in Materials and Methods using primary antibodies against showed proteins. Merge panels indicate the co-localization of antibody signals. (b) Expression of the indicated proteins was determined by Western Blot analysis. **c, d.** ER-E2F1 U2OS cells were transfected with non target siRNA (siNT) or ATP6V0C siRNAs (siATPase) and cultured in the absence or in the presence of 4-hydroxitamoxifen (OHT). (c) At 6 h after treatment, immunofluorescence assay was performed as described in Materials and Methods using primary antibodies against showed proteins. (d) Expression of the indicated proteins was determined by Western Blot analysis at the indicated times.

The above findings indicate that E2F1 mediates lysosome redistribution and increased mTORC1 signaling by inducing v-ATPase activation. Therefore we initially tested the effects of concanamycin, a specific v-ATPase inhibitor, on both responses [26]. The results show that such treatment impaired E2F1-induced redistribution of lysosomes to the cell periphery and promotes the formation of large LAMP2-positive vesicles (Figure 5a) similar to those detected in E2F1-depleted cells (Figure 1c). These vesicles may originate from the blocking of early endosome to lysosome maturation, due to the inhibition of endosomal acidification, as it has been previously described [18, 27, 28]. In parallel, the treatment with concanamycin inhibited the E2F1-induced phosphorylation of S6K1 T389 (Figure 5b). Accordingly, the knockdown of the V_0_ subunit C (ATP6V0C) of v-ATPase provoked both the formation of large LAMP2-positive vesicles and the inhibition of S6K1 T389 phosphorylation, thereby corroborating the effects obtained following concanamycin treatment (Figures 5c and 5d). Taken together, these results demonstrate that the increase in v-ATPase activity is essential for E2F1-induced mTORC1 activation and lysosomal trafficking.

### The transcriptional regulation of ATP6V0B subunit is involved in the mechanism of v-ATPase activation regulated by E2F1

**Figure 6:**
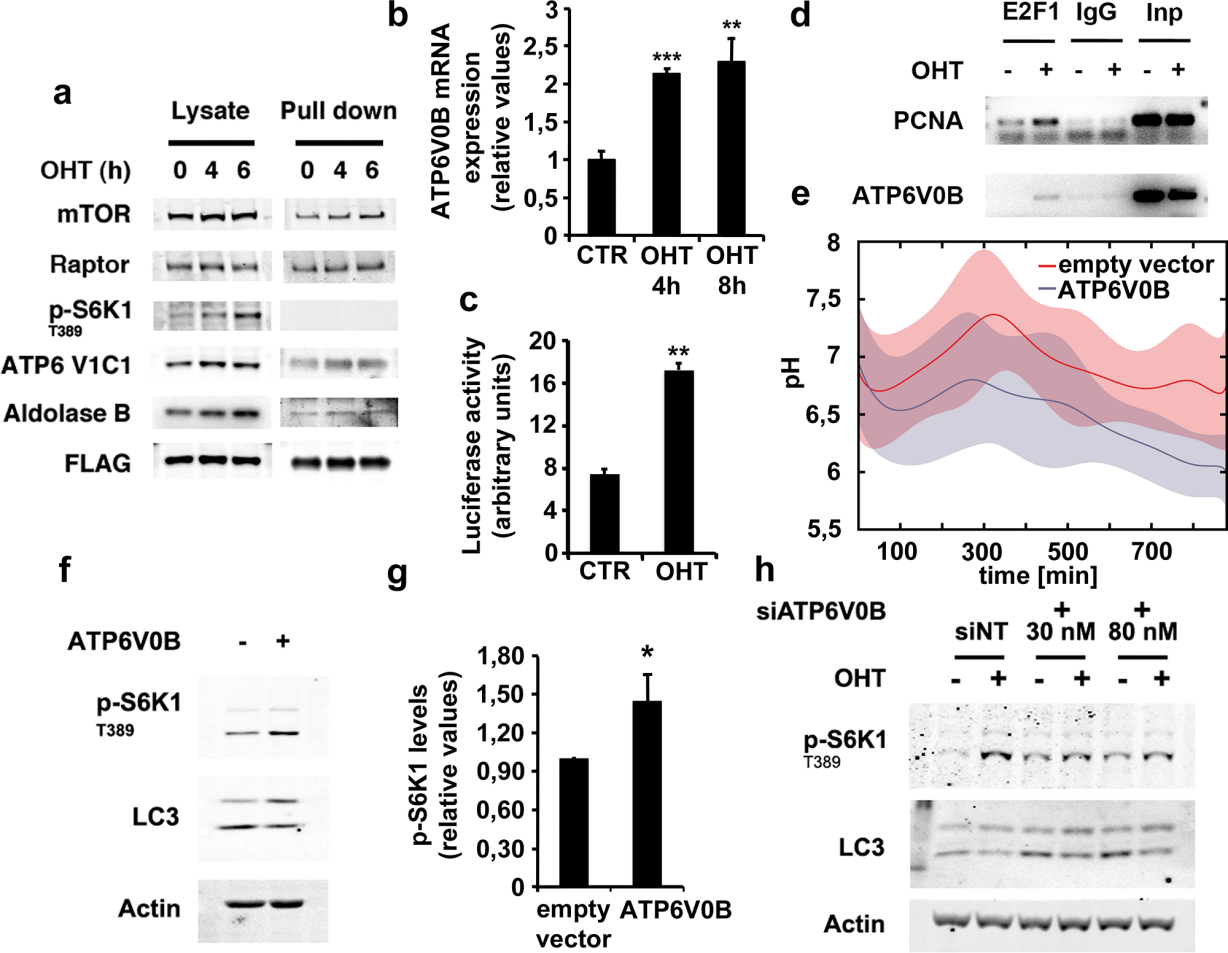
E2F1 induces ATP6V1C1 association to RagB lysosomal complex and ATP6V0B expression **a.** Serum deprived stably transfected ER-E2F1/FLAG-RagB U2OS cells were treated with 4-hydroxitamoxifen (OHT) at the indicated times. Proteins were crosslinked as described in Materials and Methods and immunoprecipitated using FLAG antibody. Expressions of the indicated proteins were determined by Western Blot analysis. **b.** Serum-deprived ER-E2F1 U2OS cells were cultured in the absence (CTR) or in the presence of 4-hydroxitamoxifen (OHT). At the indicated times, ATP6V0B mRNA levels were measured as described in Materials and Methods. **c.** ER-E2F1 U2OS cells were co-transfected with ATP6V0B-luc reporter plasmid together with CMV-renilla, serum-deprived and treated (OHT) or not (CTR) with 4-hydroxitamoxifen for 10 hours. Relative luciferase activity was calculated as the ratio of firefly and renilla activity values. Shown is mean ± S.E.M of 3 independent experiments. **d.** Serum-deprived ER-E2F1 U2OS cells were treated (+) or not (−) with 4-hydroxitamoxifen (OHT) for 6 hours. ChIP-PCR analyses of the indicated promoters were performed as described in Materials and Methods. **e.** U2OS cells were transfected with ATP6V0B expression plasmid or vector alone and then loaded with dextran labeled with FITC and with Rhodamine B for 24 h. Intra-lysosomal pH was measured as described in Materials and Methods in the presence of serum. (f) U2OS cells were transfected with ATP6V0B expression plasmid (+) or vector alone (−) as indicated. After transfection, cells were serum starved for 12 hours and expression of the indicated proteins was determined by Western Blot analysis. **g.** Quantification of S6K1 T389 phosphorylation is shown. Shown is mean ± S.E.M of 3 independent experiments. **h.** ER-E2F1 U2OS cells were transfected with non-target siRNA (siNT) or ATP6V0B siRNA at the indicated concentrations and then, serum-deprived and treated (OHT) or not (CTR) with 4-hydroxitamoxifen for 6 hours. Expression of the indicated proteins was determined by Western Blot analysis.

Next, we investigated the mechanism by which E2F1 up-regulates v-ATPase activity. It is known that, in yeast, v-ATPase activity requires the reversible association of the V_1_ domain to the membrane inserted V_0_ domain of the v-ATPase complex (Supplementary Figure S6) [29]. Given that we detected an increase in the physical interaction between mTORC1 and Rag GTPase complex after E2F1 induction (Figure 1d), we tested whether E2F1 also induced the association of the V_1_ domain to the Rag complex. To this end, we evaluated the extent of RagB interaction with the C1 subunit of the V_1_ complex (ATP6V1C1) using the U2OS ER-E2F1 cells stably transfected with a FLAG-tagged RagB. The results from FLAG immunoprecipitation experiments over 6 hours of time showed that E2F1 promoted the association of the V1 subunit C, ATP6V1C1, to RagB suggesting that, in addition to mTORC1, E2F1 is also capable of recruiting the V1 complex to lysosomes and of activating v-ATPase (Figure 6a). Aldolase, a glycolytic enzyme that has been also reported to be associated with v-ATPase, was also detected in the FLAG-tagged RagB immunoprecipitate (Figure 6a) [30]. The results indicate that the underlying mechanism by which E2F1 drives v-ATPase activation is through the recruitment of the v-ATPase subunit V1 to the RagB/mTORC1 complex.

As microarray data of expression of E2F1-regulated genes in ER-E2F1 U2OS cells are available [31], we took advantage of this information and we specifically evaluated the changes on expression of the different V-ATPase subunits after OHT treatment. Analysis of the data pointed out that the expression of ATP6V0B subunit was highly increased after E2F1 induction, a result that we confirmed by RT-PCR analysis (Supplementary Figure S7 and Figure 6b). Analysis of the ATP6V0B promoter by using an ATP6V0B-luciferase reporter vector suggested that E2F1 modulates ATP6V0B at the transcriptional level (Figure 6c) [32]. To directly prove that E2F1 regulates ATP6V0B expression through promoter binding, chromatin immunoprecipitation (ChIP) assays were performed in E2F1-activated conditions. The ChIP-PCR amplifications were performed using PCNA and ATP6V0B promoter-specific primers and demonstrated the binding of E2F1 on both target genes (Figure 6d). We further evaluated whether the expression of ATP6V0B alone was capable of regulating v-ATPase activity. Over-expression of ATP6V0B subunit induced a small, but reproducible, decrease in intra-lysosomal pH, suggesting a role for such subunit on v-ATPase activity (Figure 6e). Because of the lack of a specific ATP6V0B antibody, mRNA levels were measured as indicators of ATP6V0B expression (Supplementary Figure S8a). Consistent with the role of v-ATPase on the regulation of mTORC1 activity, ATP6V0B over-expression increased S6K1 T389 phosphorylation, while conversely, ATP6V0B depletion abrogated E2F1-induced S6K1 T389 phosphorylation (Figures 6f-6h, and Supplementary Figure S8). To confirm the role of E2F1 on ATP6V0B expression, we monitored the effect of the CDK4–6 inhibitor, Palbociclib, on ATP6V0B mRNA levels as well as on E2F1-induced phosphorylation of S6K1 T389 and 4E-BP1 T37/46. The results demonstrate that inhibition of E2F activity by impairing Rb phosphorylation, prevents the E2F1-induced ATP6V0B mRNA production as well as mTORC1 activation (Supplementary Figure S9). Overall, results here point out that expression levels of ATP6V0B subunit can regulate both v-ATPase and mTORC1 activities and that induction of ATP6V0B by E2F1 could be responsible for the effect of E2F1 on both processes.

## DISCUSSION

**Figure 7:**
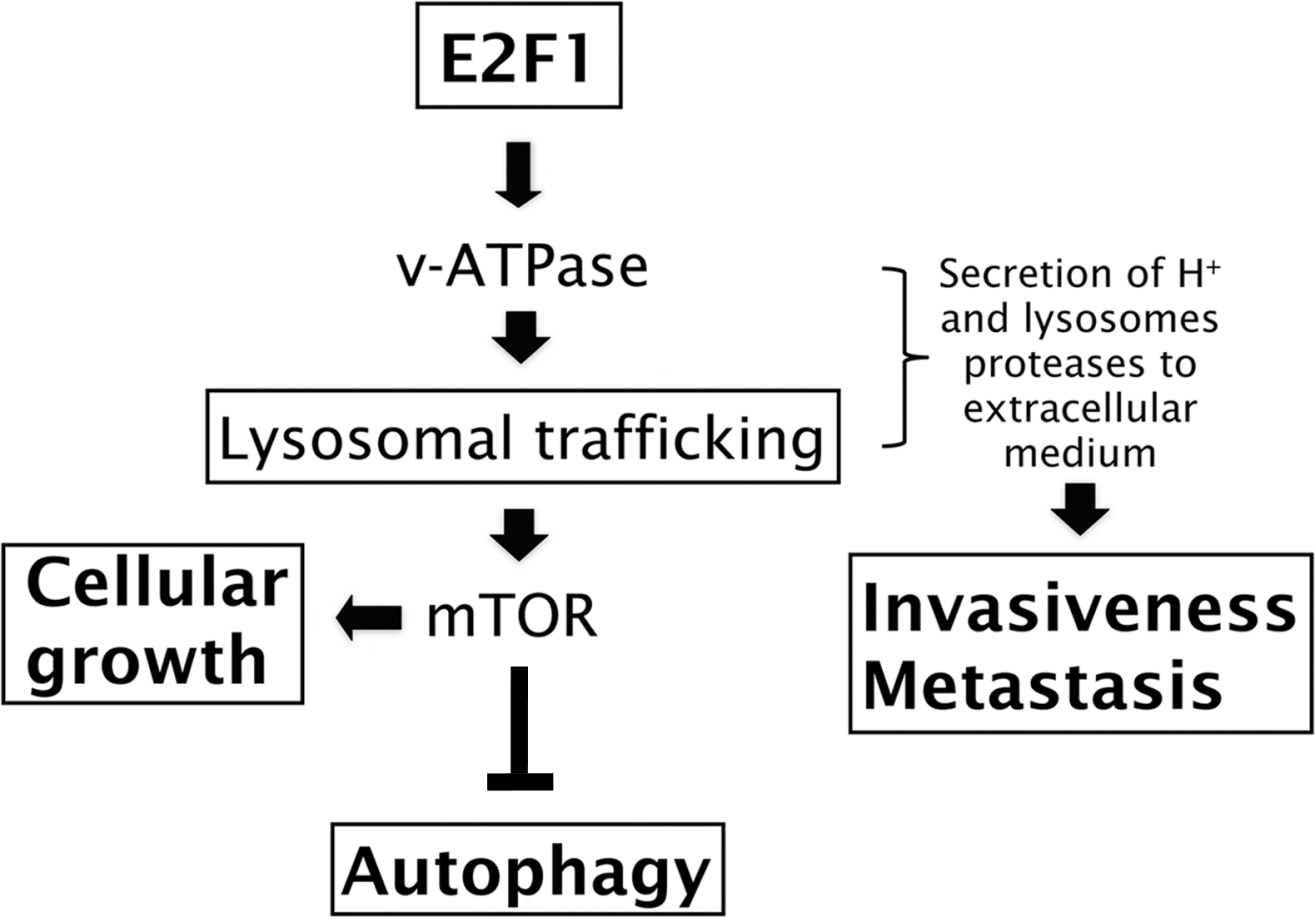
Involvement of v-ATPase on E2F1′s functions Schematic overview showing the mechanism by which E2F1 can regulate cellular growth, autophagy, invasiveness and metastasis.

Oncogenic events affect multiple intracellular signaling networks that involve interconnections and crosstalk between the individual signaling pathways. Integration of these networks defines the hallmarks that characterize tumor cells [33]. Traditionally E2F1 has been recognized as a critical regulator of cell cycle progression, through its ability to regulate the G1/S transition and S-phase entry [6, 7]. However, it has become increasingly evident that additional biological processes associated with malignant transformation are regulated by E2F1, including cell growth, autophagy, invasiveness and metastasis [2, 8–11]. In spite of the importance of these processes, the molecular mechanisms by which E2F1 modulates these responses is not extensively understood. Here, we demonstrate that E2F1 regulates the activity of v-ATPase, the major regulator of the lysosomal pH. By modulating this activity, E2F1 is capable of regulating lysosomal biology. This leads to the activation of mTORC1 and the relocalization of lysosomes to the cell periphery, required for cells to establish an acidic and active protease environment needed for promoting invasiveness and metastasis (Figure 7).

In this study, we reveal that E2F1 is able to regulate endosomal trafficking and re-direct lysosomes to the cell periphery. Localization of lysosomes at the periphery of cells has been mostly associated with invasive growth, angiogenesis and more recently, regulation of mTORC1 activity [16, 21]. Here, we demonstrate that activation of E2F1 induces mTORC1 translocation to lysosomes, where it is activated. We show that the intracellular acidification is not responsible for the peripheral movement of lysosomes induced by E2F1, as reported for the nutrient-induced lysosomal localization [21]. In contrast, E2F1 causes an alkalization of intracellular pH. Others have shown that intracellular pH of tumor cells is more elevated than untransformed controls, and importantly, this increase is sufficient to induce tumorigenicity in cultured fibroblasts [34]. It is known that E2F1 induces the transcriptional upregulation of key enzymes of the glycolytic pathway, shifting the energy production from oxidative phosphorylation to anaerobic glycolysis [35]. This produces an excess of lactate and an intracellular acidosis. Our results suggest that, in order for a cell to survive acidosis, E2F1 induces a proton-transfer from the cytoplasm to internal vesicles and to extracellular medium.

Lysosomes are not only essential for trafficking endosomal cargo but also for the autophagy process. In response to serum deprivation, lysosomes accumulate in the perinuclear area to facilitate their fusion with autophagosomes and consequently promote autophagy. This process is triggered by inhibition of mTORC1, which is essential for autophagosome formation [36]. In agreement with the action of E2F1 in driving lysosomes to cell periphery and also activating mTORC1, we demonstrated here that activation of E2F1 represses autophagy. Until now, the role of E2F1 in autophagy has been controversial. Some reports point out that E2F1 promotes autophagy by upregulating the expression of autophagy genes such as LC3, ATG1, ATG5 and DRAM [9]. On the other hand, others show that downregulation of E2F1 results in high levels of autophagy and they suggest that regulation of Bcl2 expression by E2F1 is involved in this process [10]. Our finding that E2F1 represses autophagy supports the model that genes involved in the upstream inhibition of mTORC1 signaling, including PTEN, TSC1, and TSC2, stimulate autophagy and, conversely, mTORC1-activating oncogenes, such as class I PI3K and Akt inhibit autophagy [23].

Increased E2F1 activity induced lysosomal acidification, while depletion of the endogenous transcript produced alkalization of these vesicles, confirming this response also occurs at physiological E2F1 levels. Since v-ATPase is the main contributor for maintaining the intracellular acidic milieu of lysosomes, our results imply that v-ATPase activity is modulated by E2F1 [14]. Activation of v-ATPase is associated with a number of cellular processes related to lysosomal biology, including mTORC1 regulation and endosomal/lysosomal trafficking [13, 18]. We demonstrate that inhibition of v-ATPase activity by treatment with v-ATPase inhibitor concanamycin A, or by depletion of the essential subunit for v-ATPase function, APT6V0C, abolished both processes, indicating an essential role for v-ATPase on functions regulated by E2F1 activity.

The v-ATPase holoenzyme consists of a membrane inserted V_0_ domain, which is responsible for the proton pore and a peripheral V_1_ domain responsible for ATP hydrolysis. Reversible association of V_1_ and V_0_ domains has been reported as the main mechanism of v-ATPase regulation [37]. In agreement, we demonstrated that acidification of lysosomes by E2F1 action is accompanied by the association of the C_1_ subunit of the V_1_ domain, ATP6V1C1, to v-ATPase/RagB lysosomal complex, suggesting that E2F1 could modulate v-ATPase activity through the mechanism of V_1_ and V_0_ domain reversible association. It was previously demonstrated that v-ATPase can regulate the translocation of mTORC1 to lysosomes through RagB activity modulation, as in the case of mTORC1 regulation by amino acids [13]. Here, we show that E2F1 induced an increase in the binding of mTORC1 to RagB, suggesting that once v-ATPase is activated by E2F1, RagB is needed for the of recruitment mTORC1 to lysosomes and for mTORC1 activation.

E2F1 activation induced the expression of the v-ATPase subunit, ATP6V0B, whose overexpression produced an increase in v-ATPase activity and mTORC1 activation, even though to a smaller extent compared to that of E2F1 over-expression. This indicates that other mechanisms might be involved in the regulation of both processes by E2F1. As far as we know, this is the first report demonstrating that over-expression of one v-ATPase subunit can regulate v-ATPase activity in mammals. Previously, another v-ATPase subunit, ATP6V1C1, has been described to play an essential role in the reversible assembly of the v-ATPase complex and its activation in yeast [32]. Interestingly, overexpression of the ATP6V1C1 subunit has been detected in several tumors such as oral squamous, pancreatic and hepatocellular carcinomas [38–40]. Moreover, increased ATP6V1C1 subunit expression levels were found to be associated with metastatic potential of tumors [39] and, in agreement with these findings, depletion of ATP6V1C1 subunit by siRNA resulted in the suppression of growth and metastasis in *in vitro* and *in vivo* models of hepatocellular carcinoma [40]. It will be critical to elucidate, in future studies, whether ATP6V0B could also play a similar role in tumors.

The invasive phenotype has been correlated with alteration of lysosomal trafficking and with high levels of v-ATPase activity [16]. A variety of cancer cells secrete lysosomal proteases such as cathepsins, which contribute to the degradation of extracellular matrix [41]. Thus, v-ATPase activity is necessary for acidification of the extracellular medium that allows an optimal pH for proteases to be active and induce the degradation of extracellular matrix. Our findings demonstrate that E2F1 regulates the movement of lysosomes to the cell surface, which will help to better understand the role of E2F1 in invasion and metastasis. Several studies indicated a strong association between E2F1 expression and metastasis. For example, deregulation of E2F1 enhanced invasion and metastasis of malignant melanoma without affecting proliferative activity [11]. Moreover, E2F1 is expressed at considerably higher levels in metastatic than in primary tumors of melanoma patients [11]. Importantly, both expression of E2F1 and the gene expression signature reflecting activation of E2F1 are strong predictors of progression of superficial bladder tumors to invasive tumors. From the results obtained in this study, we propose that pharmacological inhibition of v-ATPase should be tested with the aim to inhibit metastatic processes in tumors overexpressing E2F1.

## MATERIALS AND METHODS

### Cell culture and chemicals

Osteosarcoma cell line U2OS was purchased from American Type Culture Collection and stable transfected ER-E2F1 U2OS was established previously in our laboratory [8]. Cell lines were cultured in DMEM media containing 10% fetal bovine serum. Unless indicated otherwise, cells were serum-starved overnight before starting the experiments. ER-E2F1 translocation to the nuclei was achieved by incubating cells with 400 nM of 4-hydroxytamoxifen (OHT) (Calbiochem, USA). For assays using inhibitors, cells were pre-incubated for 30 minutes in serum-starved media in the presence of Rapamycin (Sigma-Aldrich, USA), BEZ235 (Novartis, Switzerland), Concanamycin A (Sigma-Aldrich, USA) and Palbociclib PD-0332991 (Selleckchem, USA).

### siRNAs, plasmids, transfection and luciferase activity

The following siRNAs were used: non-silencing CTR (GCAUCAGUGUCACGUAAUA) and ATP6V0B (GAUUUGGGCUUCCGCUUUGAU, AUCAAAGCGGAAGCCCAAAUC) siRNAs were purchased from Sigma-Aldrich (USA) ; E2F1 siRNA (sc-29297) was purchased from Santacruz Biotechnology (USA); Raptor (L-004107–00-0005) and ATP6V0C (L-017620–01-0005) siRNAs were purchased from Thermo Scientific (USA). ATP6V0B-pLX304 was purchased from DNASU (Arizona State University). LAMP1-GFP was purchased from Addgene (USA). ATP6V0B-luciferase vector was obtained by subcloning a 685 bp genomic fragment of the human gene ATP6V0B 5′ UTR-flanking region into the NheI and BglII sites of pGL3-Basic luciferase reporter vector (Promega, USA) [32]. The integrity of ATP6V0B-luc construct was confirmed by sequencing. Cells were transfected using lipofectamine 2000 according to the manufacturer instructions. For the luciferase assay, cells were co-transfected with ATP6V0B-luc and CMV-Renilla control plasmid, and luciferase and renilla activities were determined by using Dual-Luciferase Reporter Assay System according to manufacturer instructions (DLR assay system, Promega, USA).

### Quantitative real-time PCR

Following treatment, total RNA was extracted using Trizol (Invitrogen, USA). 1 μg of total RNA was subjected to reverse transcription and the resulting cDNA samples were used (diluted 1:100) in PCR amplification using LightCycler^®^ 480 SYBR Green I Master (Roche Applied Science, USA). Calculation of relative mRNA was done using Light Cycler 480 software. The following primers were used: ATP6V0B, 5′-ATCATCTTCTGTGAGGCTGTGGC-3′ (forward), and 5′-AGACTCCACAGAAGAGGTTAG ACAG-3′ (reverse); Actin, 5′-AATGTGGCCGAGGACTTTGATTG C-3′ (forward), and 5′-AGGATGGCAAGGGACTTCCT GTAA-3′ (reverse).

### Western blot

Protein extraction, separation and detection were achieved as described before [8]. The following antibodies were used: anti-human E2F1 (1:500), anti-S6K1 (1:3000) and anti-Aldolase B (1:1000) from Santacruz Biotechnology (USA), anti-LC3 (1:2000) from MBL International (Germany), anti-FLAG (1:5000) from Sigma Aldrich (USA), anti-ATP6V1A (1:2000) from Thermo Scientific (USA), anti-ATP6V1C1 (1:1000) from Aviva Systems Biology (USA), anti-raptor (1:1000) from Millipore (USA), anti-pS6K1 (1:2000), anti-p4EBP1 (1:3000), anti-mTOR (1:1000), anti-pRb (1:1000) and anti-actin from Cell Signaling Technology (USA).

### Live-cell imaging analysis of lysosomal trafficking

ER-E2F1 U2OS cells were plated in 8 wells chamber slide (Ibidi) and transiently transfected for 24 h to express LAMP1-GFP. After overnight serum starvation, cells were treated as described. Fluorescence was analyzed on the Leica TCS SP5 spectral Live confocal microscope using a 63X N.A 1.4 objective and LAS AF software, within an incubation chamber XL LSM710 S1 (PeCon GmbH, Germany) with a heating insert P-LabTek S1. GFP fluorophore was excited with Argon laser 15%. Time-lapse images were taken from six regions for each sample every minute for 20 hours. Images were analyzed with ImageJ software and converted into avi format to be edited with Final Cut software.

### Measurement of lysosomal pH in live cells

ER-E2F1 U2OS cells were plated in 8 wells chamber slide (Ibidi) and loaded with a 70, 000-Da dextran that was coupled to FITC and to Rhodamine (Fluorescein isothiocyanate–dextran, Rhodamine B isothiocyanate Dextran) (Sigma-Aldrich, USA) at 1 mg/ml during 20 hours in serum starved conditions. After washing, cells were treated as indicated. Fluorescence was analyzed on the Leica TCS SP5 spectral Live confocal microscope using a 63X N.A 1.4 objective and LAS AF software. Time-lapse images were taken from six regions for each condition every minute during 20 hours using excitation wavelengths of 488 for FITC and 568 for Rhodamine. The fluorescence intensity of the images was analyzed using ImageJ software. As a result, the red and green signals as a function of time were obtained. These curves featured a noisy pattern, so that each of them was smoothened by a polynomial interpolation. The green-to-red ratio was computed from each of these smoothened curves and then from these an average curve with standard deviation was computed for all six positions. Calibration was performed by incubation of the cells with media adjusted between 5.0 to 8.0 pH values containing 10 **μ**M of nigericin (Panreac Sciences, Spain) and 10 **μ**M Valinomycin (Sigma-Aldrich, USA).

### Measurement of intracellular pH

Intracellular pH was measured as previously described [42]. Briefly, ER-E2F1 U2OS cells were treated as described, resuspended in PBS and loaded with 5 μM of 5-(and 6)-Carboxy SNARF-1 Acetoxymethyl Ester (SNARF-1, AM) (Molecular Probes, USA) for 30 min at 37 **°**C. Finally, the cells were washed and run on a MoFlo Astrios Cell Sorter (Beckman Coulter, USA) at an excitation wavelength of 488 nm and an emission of 576 nm and 664 nm using linear amplification. The ratio 664/576 was determined to normalize the fluorescence intensity. Calibration curve was performed by incubation of the cells with high-K^+^ calibration buffers adjusted between 6.0 to 8.0 pH values containing 10 μM of Nigericin (Panreac Sciences, Spain).

### Immunofluorescence analysis

Cells were plated onto glass coverslips and procedure was achieved as described previously [8]. The following antibodies were used: mTOR rabbit monoclonal (dilution 1:150) from Cell Signaling Technology (USA); LAMP2 mouse monoclonal (dilution 1:300) from BD Biosciences (USA); LC3 rabbit polyclonal (dilution 1:300) from MBL International (Germany).

Fluorescence was detected with the Leica spectral confocal microscope TCS SP5 using a 63X N.A 1.4 objective and LAS AF software. Fluorophores were excited with Argon laser 15% for 488 nm, DPSS 561 for 555 nm and Diode laser for 405 nm. Images were analyzed with ImageJ software.

### Cross-linking and immunoprecipitation

After the treatment, U2OS cells stably expressing FLAG-RagB and ER-E2F1 were washed twice with ice-cold PBS and incubated for 7 minutes at room temperature with 1 mM DSP crosslinker reagent in PBS (Thermo Scientific, USA). 1 M Tris-HCl (pH 7.5) was added 1:10 to quench DSP and cells were washed twice prior to lysis in ice-cold RIPA buffer (1% NP-40, 1% deoxycholate, 0.1%SDS) in the presence of protease and phosphatase inhibitors cocktails (Sigma Aldrich, USA). The soluble fractions from cell lysates were isolated by centrifugation at 13, 000 rpm for 5 minutes in a microfuge. For immunoprecipitations, 30 μl of a 33% slurry of anti-FLAG M2 beads (Sigma Aldrich, USA) was added to each lysate and incubated with rotation overnight at 4°C. Immunoprecipitates were washed three times with RIPA buffer supplemented with 500 mM NaCl and once with normal RIPA buffer. Immunoprecipitated proteins were resolved by 4%–20% SDS-PAGE.

### Migration assay

Cell migration was dynamically recorded using the xCELLigence RTCA-DP system (Roche, Germany) following the manufacturer instructions. 25, 000 cells were plated in the upper chamber coated with collagen-type I and the migration into the bottom chamber, in response to fetal serum as a chemoattractant, was monitored during 24 hours. The electrical impedance caused by the migration of the cells from the upper chamber to the lower chamber is converted to Cell Index Value. Thus, the Cell Index is an indicator of the cell capacity to invade the matrix and migrate. The RTCA Software 1.2 was used to analyze the data.

### Chromatin immunoprecipitation assays

Chromatin immunoprecipitation assays were performed following the Standard ChIP protocol from Millipore. Briefly, cells were treated with formaldehyde (final concentration 1%) to crosslink chromatin and harvested. Cell lysates were then prepared and sonicated on ice to break chromatin DNA to an average length of 500 bp. Cell lysates (from 4 × 106 cells per sample) were incubated with 2 μg of E2F1 antibody (KH95, Santa Cruz Biotechnology). DNA amplification of the captured DNA immunocomplex was performed using primers complementary to the ATP6V0B and PCNA-promoter regions harboring putative E2F1-binding sites. The primer sequences were 5′-AGCGTCGCCTTGCTCTAGAC-3′ as forward and 5′-AAGACCCCGGAGTAGAGCAGTG-3′ as reverse to generate a 228 bp amplification product for ATP6V0B promoter; 5′-TTCTCATTGGCCTGCCACGC-3′ as forward and 5′-ACCACCCGCTTTGTGACTTTG-3′ as reverse to generate a 117 bp amplification product for PCNA promoter. DNA samples were analysed by electrophoresis on 2% agarose gels, stained with ethidium bromide and then photographed by using a Gel Doc System.

### Statistic analysis

Data was analyzed by GraphPad Prism4 software. Results were presented as Mean (S.E.M for *n* = 4. Experimental data sets were compared by a two-sampled, two-tailed and unequal S.D. Student *t* Test. Values of *P(0.05, **P(0.005 and ***P(0.0005 were considered statistically significant.

† In memory of Dr. Josep Carreras i Barnés

## SUPPLEMENTARY INFORMATION FIGURES AND MOVIES


