# Supplementary material for: V-ATPase: a master effector of E2F1-mediated lysosomal trafficking, mTORC1 activation and autophagy
Source: Oncotarget. 2015 Aug 11;6(29):28057–70. doi: 10.18632/oncotarget.4812 (PMC4695044; doi:10.18632/oncotarget.4812)
Supplement: Supplementary file 1 [file oncotarget-06-28057-s001.pdf]

# SUPPLEMENTARY INFORMATION

## REFERENCES

1. Kane PM, Parra KJ. Assembly and regulation of the yeast vacuolar H(+)-ATPase. The Journal of experimental biology. 2000; 203:81–87.
2. Marshansky V, Futai M. The V-type H+-ATPase in vesicular trafficking: targeting, regulation and function. Current opinion in cell biology. 2008; 20:415–426.
3. Shats I, Gatza ML, Liu B, Angus SP, You L, Nevins JR. FOXO transcription factors control E2F1 transcriptional specificity and apoptotic function. Cancer research. 2013; 73:6056–6067.

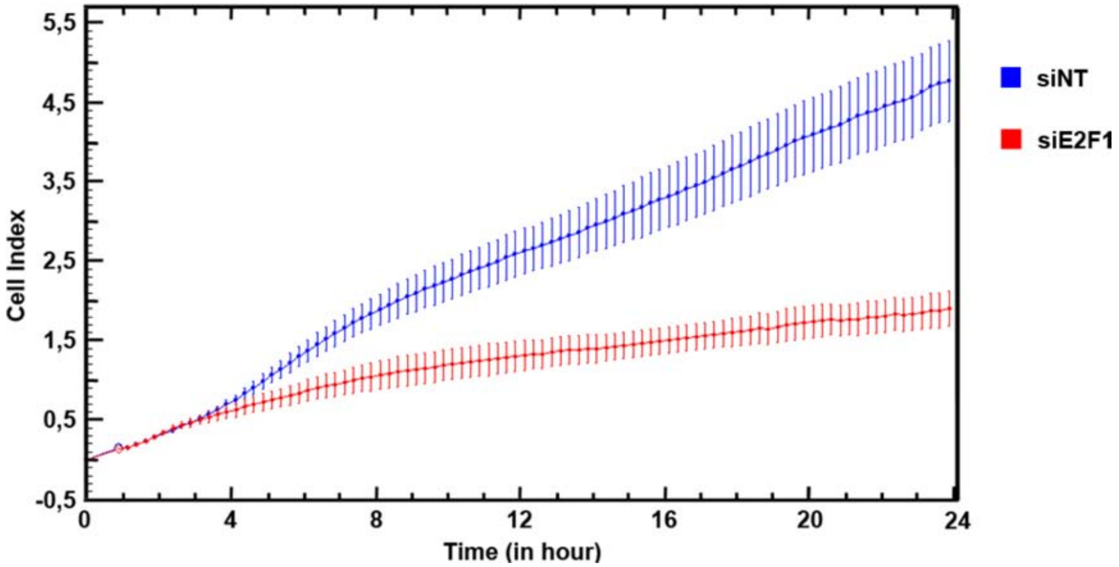


**Supplementary Figure S1: Role of E2F1 in cell migration.** U2OS cells were transfected with 30 nM of non-target siRNA (siNT) or E2F1 siRNA (siE2F1) as indicated. Cell migration was dynamically recorded using the xCELLigence RTCA-DP system as described in Materials and Methods.

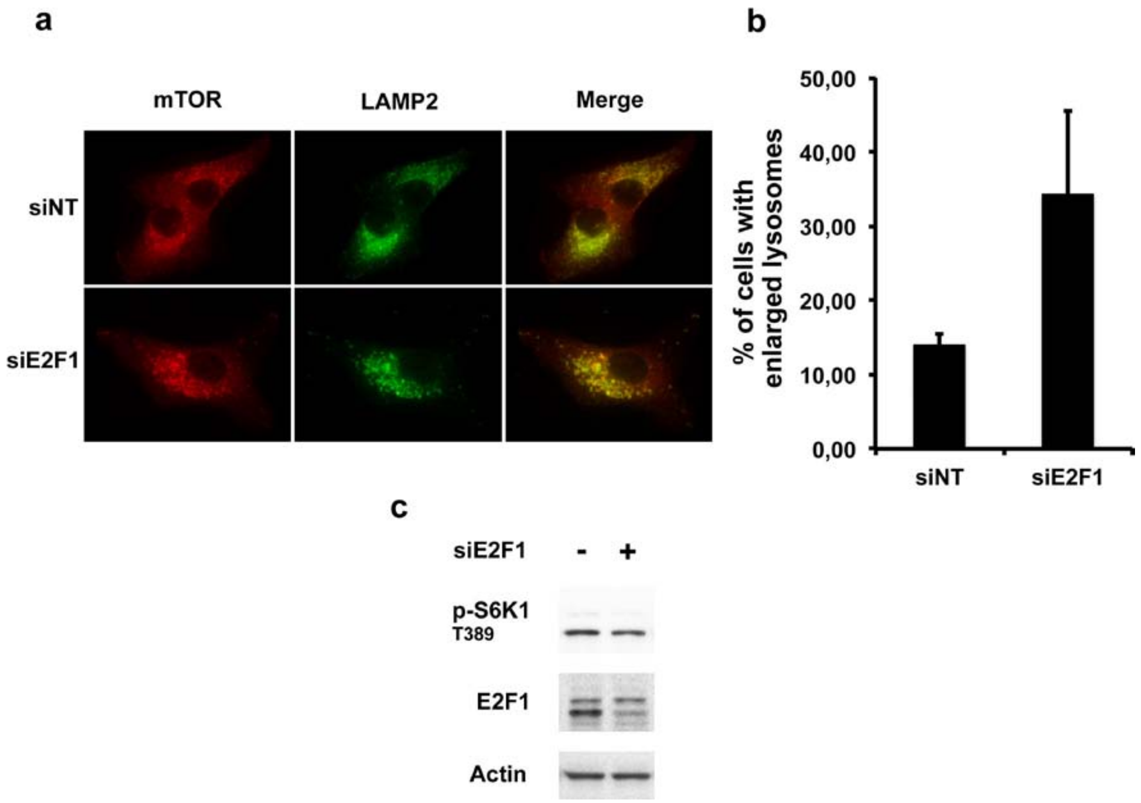


**Supplementary Figure S2: Effect of E2F1 depletion in A549 cell line. a–c.** A549 cells were transfected with non target siRNA (siNT) or E2F1 siRNA as indicated. Cells were serum-deprived and immunofluorescence was performed as described in Materials and Methods using primary antibodies against specified proteins at 48 h after transfection; (b) Quantification of enlarged lysosomes is shown (c) Expressions of the indicated proteins were determined by Western Blot analysis.

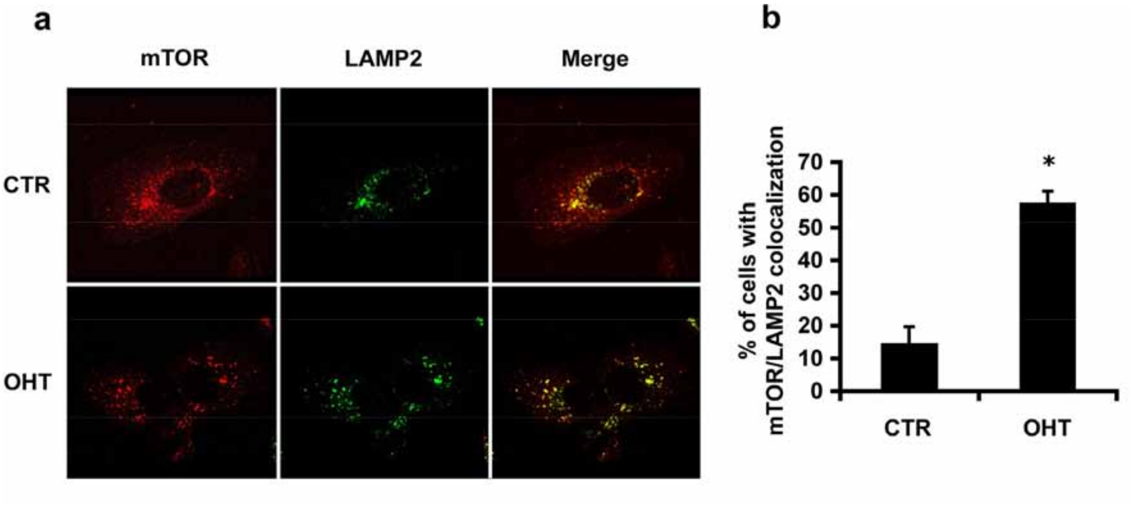


**Supplementary Figure S3: E2F1 increases mTOR co-localization with LAMP2 marker. a, b.** Serum-deprived ER-E2F1 U2OS cells were cultured in the absence (CTR) or in the presence of 4-hydroxitamoxifen (OHT). At 6 h after treatment, immunofluorescence assay was performed as described in Materials and Methods using primary antibodies against showed proteins. Merge panels indicate the co-localization of antibody signals. (b) Quantification of % of cells with mTOR/LAMP2 co-localization is shown. Shown is mean ± S.E.M of 3 independent experiments.

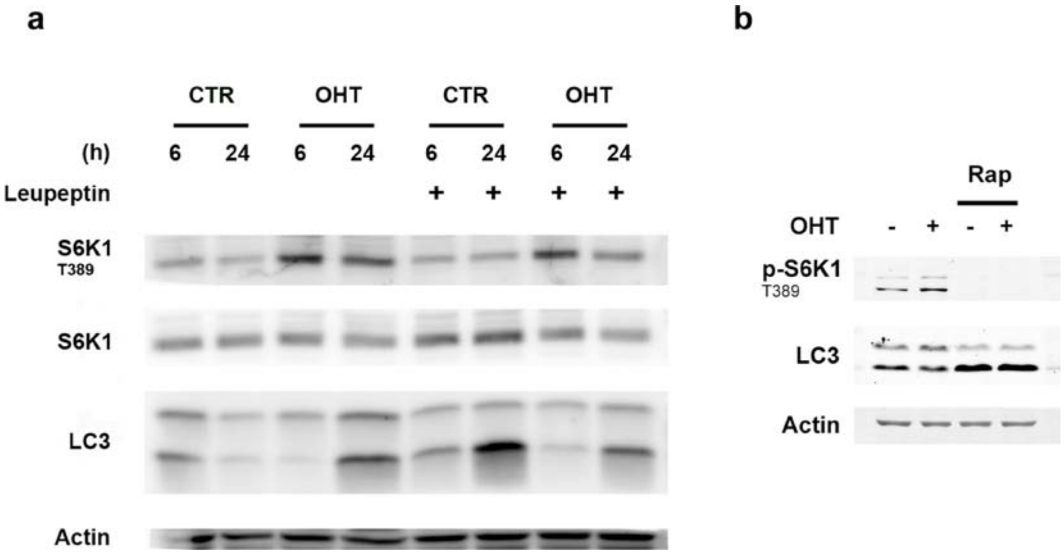


**Supplementary Figure S4: Effect of E2F1 activation on autophagy. a.** Serum-deprived ER-E2F1 U2OS cells were cultured in the absence (CTR) or in the presence of 4-hydroxitamoxifen (OHT). At showed times, expression of the indicated proteins was determined by Western Blot analysis in the presence (+) or in the absence of leupeptin. **b.** Serum-deprived ER-E2F1 U2OS cells were cultured and treated (+) or not (−) with OHT in the presence or in the absence of rapamycin (Rap). Expression of the indicated proteins was determined by Western Blot analysis at 6 h after treatment.

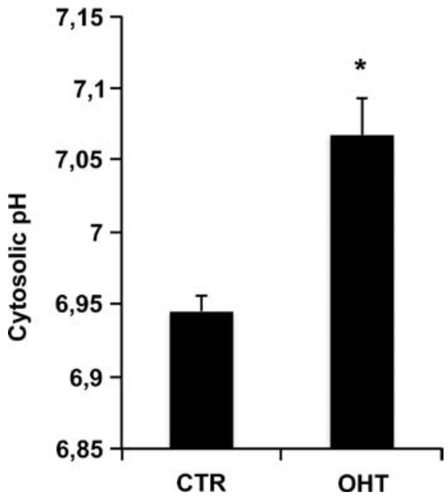


**Supplementary Figure S5: Effect of E2F1 activation on intra-cellular pH.** ER-E2F1 U2OS cells were serum-starved, cultured in the absence (CTR) or in the presence of 4-hydroxitamoxifen (OHT) and incubated with SNARF-1 dye for 30 minutes. At 20 h after treatment, intracellular pH was measured as described in Materials and Methods. Shown is mean ± S.E.M of 2 independent experiments.

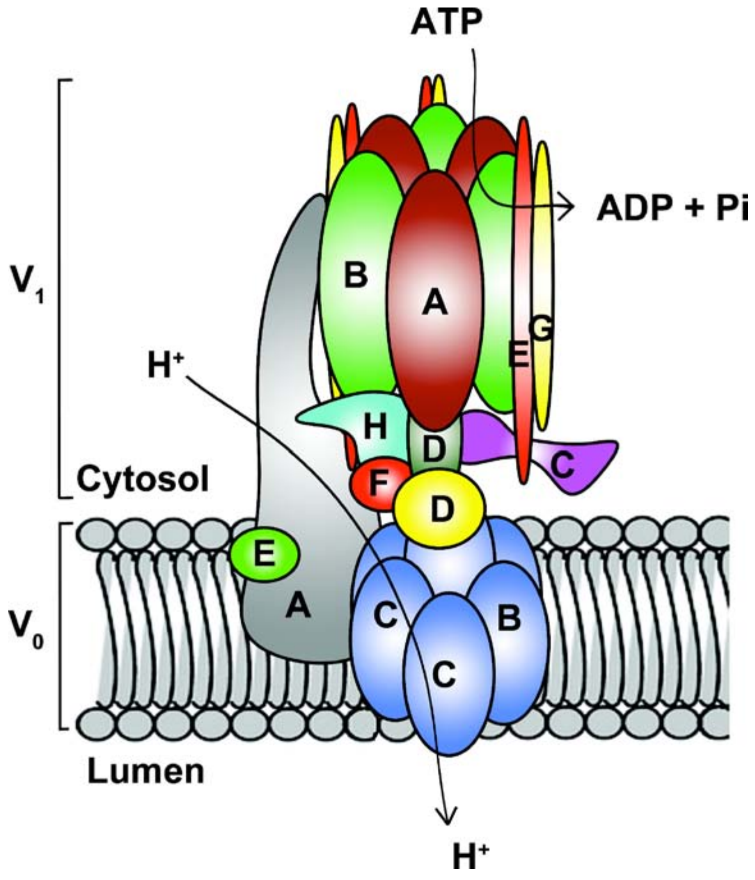


**Supplementary Figure S6: Schematic V-ATPase structure.** Adapted from yeast v-ATPase structure[1, 2].

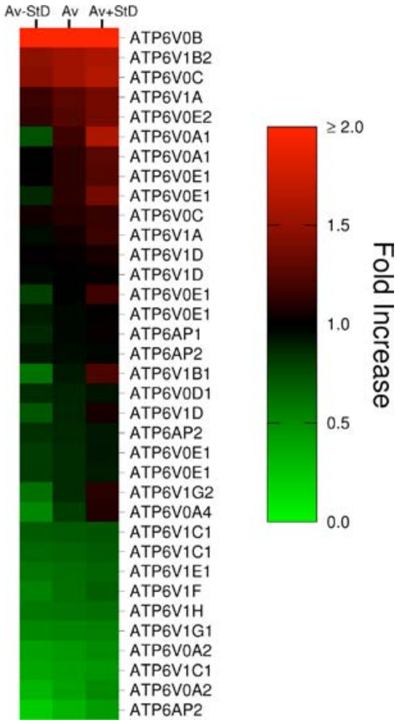


**Supplementary Figure S7: Heatmap of v-ATPase subunits gene expression regulated by E2F1.** Microarray data of E2F1-induced genes in ER-E2F1 U2OS cells are public available [3]. Analysis of the v-ATPase subunits expression profile was performed as following: The average fold increase (Av) for each gene was calculated among the 16 possible combinations of the ratio between 4 experimental repetitions of the 4-hydroxitamoxifen/control conditions. The relative magnitude of expression of the E2F1-activated genes is indicated on a spectrum ranging from minimum (green) to the maximum detected (red). Red color indicates up-regulation, and green color indicates down-regulation, of gene expression relative to the control. (Av − StD) and (Av + StD) values represent the average plus and minus standard deviation.

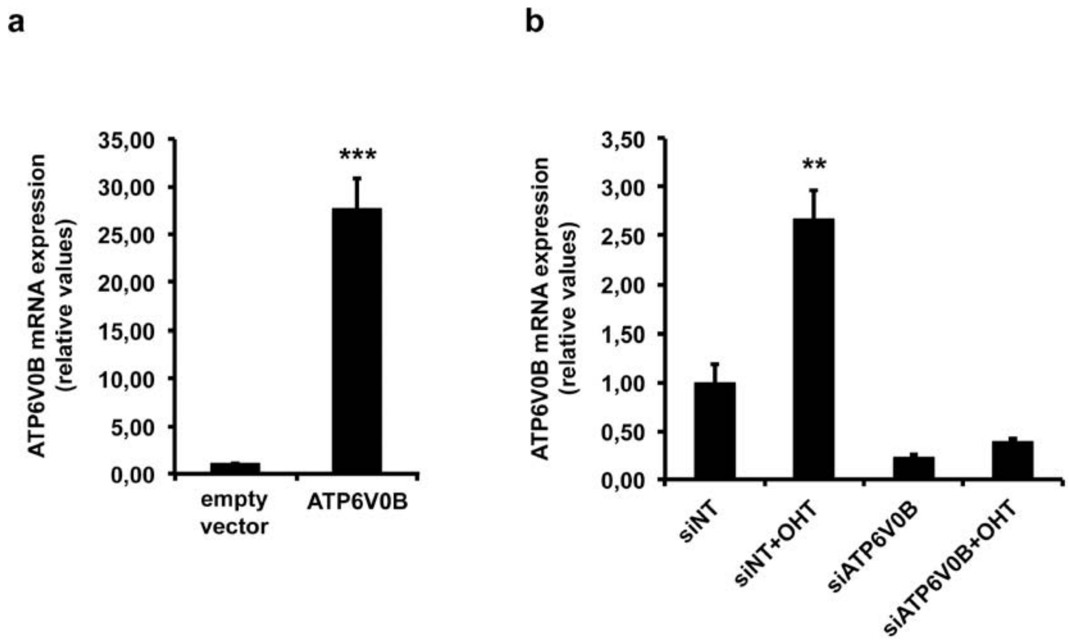


**Supplementary Figure S8: ATP6V0B mRNA levels in ATP6V0B over-expression and knockdown experiments. a.** U2OS cells were transfected with ATP6V0B expression plasmid or empty vector as indicated in Figure 6f. mRNA levels were measured by real-time PCR as described in Materials and Methods. Shown is mean ± S.E.M of 3 independent experiments. **b.** ER-E2F1 U2OS cells were transfected with 80 nM of non-target siRNA (siNT) or ATP6V0B siRNA and treated as indicated in Figure 6h. mRNA levels were measured by real-time PCR as described in Materials and Methods. Shown is mean ± S.E.M of 3 independent experiments.

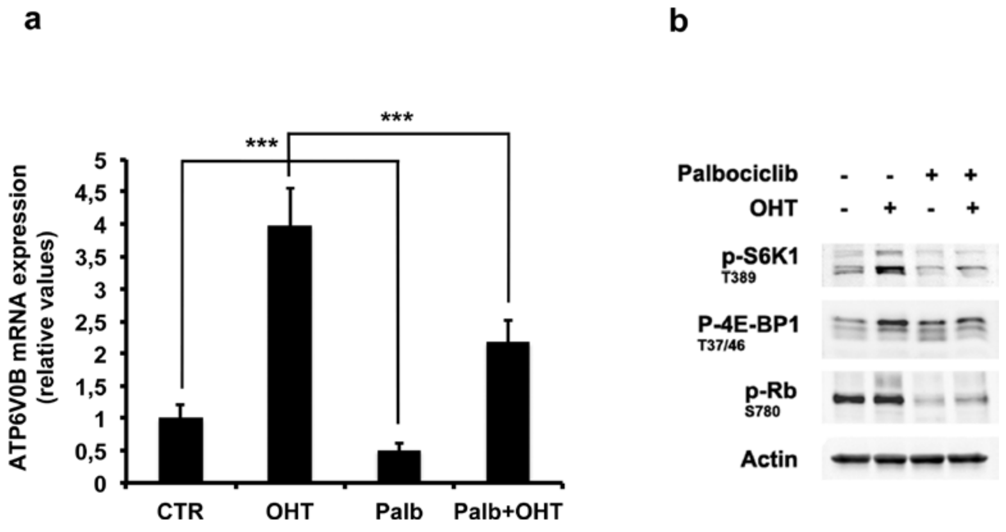


**Supplementary Figure S9: Effect of palbociclib on E2F1-induced mTORC1 activity. a, b.** Serum-deprived ER-E2F1 U2OS cells were treated (+) or not (−) with 4-hydroxitamoxifen (OHT) for 6 hours in the absence or presence of 1 μM Palbociclib (Palb). (a) ATP6V0B mRNA levels were measured as described in Materials and Methods. (b) Expression of the indicated proteins was determined by Western Blot analysis.

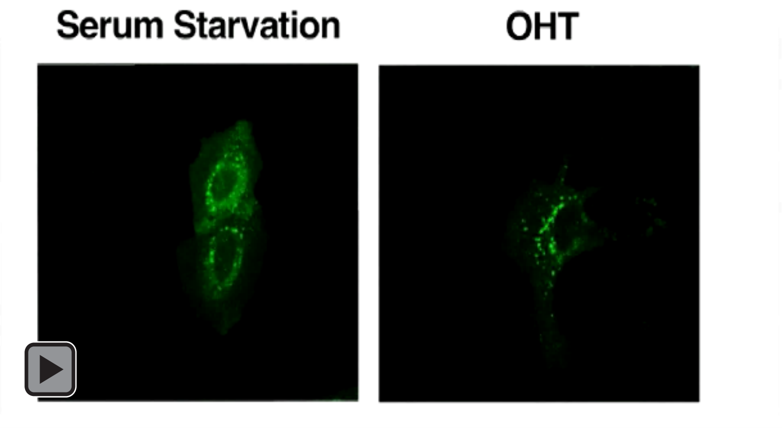


**Supplementary Movie S1: Live-cell imaging of lysosomal movement induced by E2F1.** ER-E2F1 U2OS cells were transfected with GFP-LAMP1 for 24 h. Cells were then serum-deprived for 15 h and cultured in the absence (CTR) or in the presence of 4-hydroxitamoxifen (OHT). Live-cell imaging was achieved as described in Materials and Methods. Time-lapse fluorescence movie shows a cell expressing GFP-LAMP1 protein.

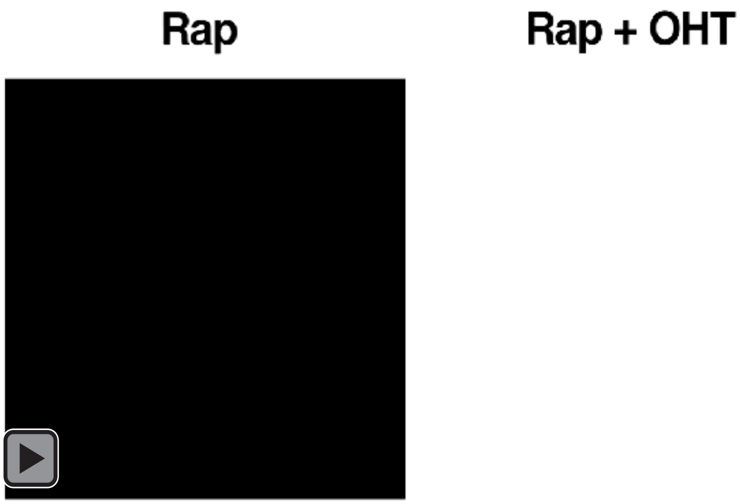


**Supplementary Movie S2: Live-cell imaging of the effect of rapamycin on the lysosomal movement induced by E2F1.** ER-E2F1 U2OS cells were transfected with GFP-LAMP1 for 24 h. Cells were then serum-deprived for 15 h and cultured in the absence (CTR) or in the presence of 4-hydroxitamoxifen (OHT) with or without rapamycin (Rap). Live-cell imaging was achieved as described in Materials and Methods. Time-lapse fluorescence movie shows a cell expressing GFP-LAMP1 protein.
